# Elimination of Cancer Health Disparities through the Acceleration of HPV Vaccines and Vaccinations: A Simplified Version of the President’s Cancer Panel Report on HPV Vaccinations

**DOI:** 10.4172/2157-7560.1000361

**Published:** 2017-05-29

**Authors:** Eva McGhee, Hill Harper, Adaku Ume, Melanie Baker, Cheick Diarra, John Uyanne, Sebhat Afework, Keosha Partlow, Lucy Tran, Judith Okoro, Anh Doan, Karen Tate, Mechelle Rouse, Meidrah Tyler, Kamilah Evans, Tonya Sanchez, Ishmum Hasan, Enijah Smith-Joe, Jasmine Maniti, Liliana Zarate, Camille King, Antoinette Alugbue, Chiamaka Opara, Bileko Wissa, Joanne Maniti, Roland Pattillo

**Affiliations:** Department of Internal Medicine, Charles Drew University of Medicine and Science, Los Angeles, USA

**Keywords:** Cancer, HPV vaccine, Gardasil, Cervarix, Gardasil-9

## Abstract

The human papillomavirus (HPV) is a major public health concern affecting both females and males. HPV is associated with cervical, anal, head and neck cancers. About 99% of all cervical cancers are related to HPV. HPV vaccines, Gardasil, Cervarix, and Gardasil 9 are used in the primary prevention of HPV related cancers. Gardasil and Gardasil 9 are available for use in both females and males ages 9 to 26, while Cervarix is available for females ages 9 to 25. Gardasil 9 was approved by the FDA for prevention against additional HPV types. Despite the availability of this preventative measure against cervical cancer, the rate of HPV vaccination in the United States remains lower than that of other industrialized nations. The purpose of this study is to elucidate mechanisms to help increase the HPV vaccination rate by using education as a tool; by simplifying the president report so that lay person can understand the information presented in the report. Through the quantitative examination of the data from the states with the lowest and highest vaccination rates, using SPSS statistical analysis; we analyzed several factors involved with the low uptake of the vaccines. The results collected show that socioeconomic status, misconceptions about HPV, and misconceptions about the safety of the vaccines were identified as possible obstacles to the effective uptake of HPV vaccinations. The proposals made by the President’s Cancer Panel to accelerate the uptake of vaccines include, increasing coverage of the vaccines through government-sponsored programs, and the Affordable Care Act; increasing accessibility to vaccines through pharmacies, schools, and clinics; and disseminating more information on HPV to healthcare providers, parents, caregivers, and patients. Allowing greater accessibility to the vaccines for all populations regardless of income, education, and eliminating misconceptions of the vaccines would play a significant role in eliminating cancer.

## Introduction

**Figure 1:**
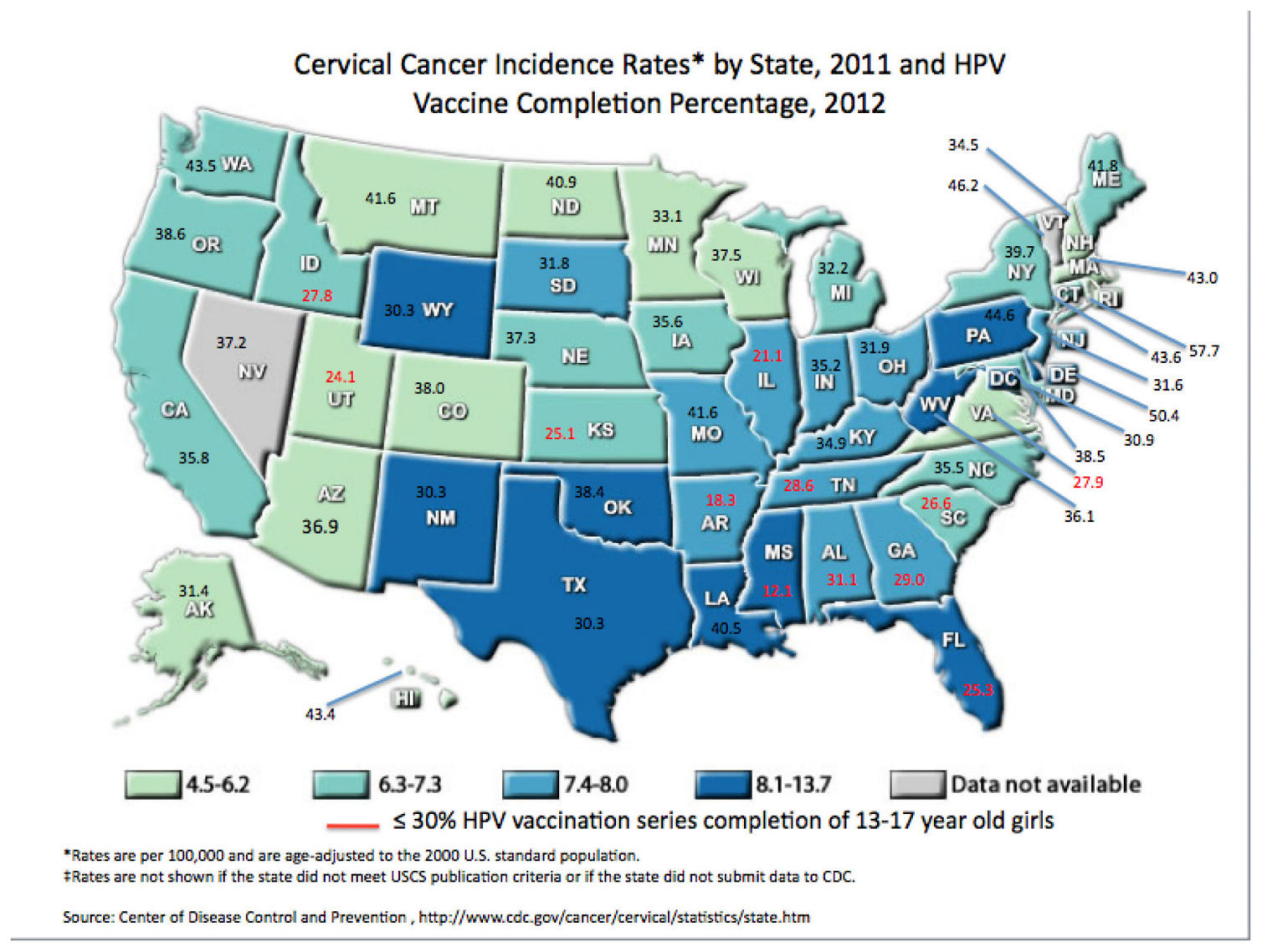
Percentage of 13 to 17 Year-Old Girls Completing HPV Vaccines Series, and Cervical Cancer Incidence Rates by State, and HPV Vaccine Completion Percentage Map. HPV vaccine completion percentages are displayed in numerical value to their corresponding states. The colors represent the cervical cancer incidence rate bracket each state falls in. States with less than or equal to 30 percent vaccine completion rates are highlighted in red.

The Human papillomavirus (HPV) infection is a major public health concern. Approximately 80 million or one in four people are infected with this virus in the United States [1]. HPV infections cause nearly 26,000 new cases of cancer in the U.S. and more than 600,000 cases worldwide each year [2]. Despite the availability of HPV vaccines that serve as primary prevention against these cancers, there is evidence that show low rate of uptake as low as 12% in some states in US and the highest uptake in the US being almost 57.7% (Figure 1 and Table 1) also shown in Figure 1 is the cervical cancer incidence rates by states [3]. This finding alarmed the President’s Cancer Panel, and became the focus of the Panel’s annual 2012–2013 report. In the report, the panel claims that HPV vaccine uptake lags behind that of other adolescent vaccines, leaving millions of adolescents vulnerable to infection with the cancer-causing virus [3]. The panel lists four goals in the report that if met, would ultimately lead to higher rates of vaccine uptake and significantly lower rates of cervical cancer and other cancers in the future. Although the Panel’s charge is focused on the U.S. National Cancer Program, the Panel recognizes the role of the United States in supporting cancer control efforts in other parts of the world. The Cancer Panel hopes that if HPV vaccination is made a public health priority by many different organizations, HPV and it’s associated diseases would be eradicated [3,4].

### HPV is a sexually transmitted infection that is linked to the occurrence of various cancers

The Human Papillomavirus (HPV) consists of a group of over 200 types of double-stranded DNA viruses belonging to the Papillomaviridae family. Genital human papillomavirus (HPV) is the most common sexually transmitted infection in the United States. More than 40 types of HPV are typically transmitted through sexual contact, and infect the anogential region (anus and genitals) [4]. Most HPV infections do not present definite or readily observable symptoms; however, some infections progress to develop benign papillomas (warts). HPV is known to infect keratinocytes in the skin and mucosal membranes and can cause abnormal growth of tissue, leading to precancerous lesions and invasive cancers of the cervix, vulva, vagina, penis, oropharynx, or anus [5]. “High-risk HPV infection” is the cause of nearly all cases of cervical cancer. An estimated 14 million people are newly infected with HPV each year; approximately half of the new HPV infections occur among people aged 15–24 years this data represents the US and foreign countries; HPV accounts for considerable morbidity and economic burden in the US population [6].

Annual costs of cervical cancer screening and treatment of HPV-associated conditions have been estimated at $8 billion U.S. dollars. In 2009, approximately 30,000 HPV-associated cancers were reported in the United States, with 39% prevalence in males Table 2. As previously mentioned, over 200 HPV types have been identified, with HPV types 16 and 18 causing approximately 70% (400,000 reported cases) of cervical cancers globally [3,7].

### HPV vaccines prevent the occurrence of cervical cancer and other HPV-associated health outcomes

Three HPV vaccines are licensed in the United States for prevention of specific HPV types and HPV-associated outcomes. Gardasil^®^ (Merck and Co., Inc.), a quadrivalent HPV vaccine, was licensed by the Food and Drug Administration (FDA) in 2006 for use in females aged 9–26 years as prevention of cervical cancer and its precursors, vulvar and vaginal cancer precursors, and anogential warts caused by HPV types 6, 11, 16 and 18 [3]. Three years later, the FDA licensed the bivalent vaccine, Cevarix^®^ (GlaxoSmithKline) for use in females aged 9–25 years for prevention of cervical cancer and its precursors caused by HPV types 16 and 18. The FDA approved Gardasil 9^®^ (Merck and Co., Inc.), a 9-valent HPV vaccine for prevention against HPV types 6, 11, 16, 18, 31, 33, 45, 52, and 58 in December 2014 Table 3 [8]. Gardasil 9 is a vaccine approved for use in females in the same age range recommended by the quadrivalent Gardasil vaccine and is also recommended for use in male’s ages 9 through 15. The 9-Valent vaccine (Gardasil 9) has the potential to prevent approximately 90% of cervical, vulvar, vaginal and anal cancers. Both vaccines are administered as a 3-dose series over 6 months. The 9-valent HPV Vaccine was introduced in 2014 as one of his newest forms of cancer prevention among males and females between the ages of 9 to 26 years [9,10]. Researchers of this vaccine tested the efficacy and immunogenicity among women age 16–26 in a randomized, international, double-blinded test. Researchers ultimately found that this vaccine offered the potential to increase overall prevention of cervical cancer from 70% to 90%. there were limitations within the study, the FDA felt that the results of the study were significant enough to release the drug to the public [11].

HPV vaccines work by stimulating the body to produce antibodies that recognize and bind to the HPV virus, preventing it from infecting cells. The current HPV vaccines are made up of virus-like particles (VLPs) that have been formed from HPV surface components using recombinant DNA technology [12]. Because the VLPs lack viral DNA, they are not infectious. However, since they contain the same surface components as the actual HPV virus, the antibodies produced from the vaccine will recognize the natural virus. This makes the vaccines highly effective.

### HPV vaccine uptake has not kept pace with that of other adolescent vaccines over the years

Today, there are three effective vaccines that prevent infection by the two most prevalent cancer-causing HPV types. However, in 2012, 33% of adolescent females and 7% of adolescent males across the U.S. completed the three-dose series [13]. These low vaccination rates reveal countless missed opportunities to prevent cancers and other HPV-associated health outcomes. HPV vaccines are underused not only in the U.S., but also around the world. The President’s Cancer Panel’s Annual Report (2012–2013) addresses this concern about the HPV vaccine under-use which poses a serious threat to progress against cancer and notes specific goals in order to increase vaccine uptake. These goals being to reduce missed clinical opportunities, increase acceptance of disease, maximize access to the vaccine and promote global vaccination uptake. A 2010 study on HPV adherence shown that very few females receive their second and third vaccinations early, many of them receive it late. It was also determined that there were lower completion rates among African American patients compared to their white counterparts [3,14].

### President Obama’s Cancer Panel addresses concerns over vaccine underuse in 2012–2013 report

In the United States, rates of cervical infection with HPV types covered by the vaccines fell by more than 50% among girls 14–19 years in the four years following vaccine introduction. The decline was even more dramatic across the globe, as the prevalence of HPV infections covered by the vaccine among females under the age of 21 plummeting by as much as 90%. Although 56 million vaccine doses have been administered in the United States as of early 2013, The U.S. Advisory Committee on Immunization Practices (ACIP) and the President’s Cancer Panel called for “urgency for action” in accelerating the HPV vaccine uptake in the 2012–2013 annual report to President Obama [3,15]. The panel’s report urges for the safety and efficacy of these vaccines through “excellent safety profiles” similar to those of other licensed adolescent vaccines, citing no serious safety concerns. The U.S. ACIP recommends routine vaccination of females ages 11 or 12 years with the three doses of either Cervarix^®^ or Gardasil^®^. The vaccination series can be started beginning at age 9 years. Vaccination is recommended for female’s ages 13–26 who have not been vaccinated previously or who have not completed the three-dose series.

In 2007, the first full year after Gardasil was approved in the U.S., about 25% of 13 to 17 years old girls received at least one HPV vaccine dose, with this rate being similar to the proportion that received other adolescent vaccines during the first year they were recommended. In 2012, 53.8% of 13 to 17-year-old girls had received the first HPV dose, with 33.4% completing all three recommended doses.

The panel’s report pleads for HPV vaccine uptake to be a public health priority and lists a plan of action by recruiting for help from the government, providers, parents and adolescents. The panel recommends the following four goals to help increase uptake of HPV vaccines in the United States (and globally) with hopes of having age-eligible adolescents fully vaccinated (completion of the three-dose vaccine).

### Goal 1: Reduce Missed Clinical Opportunities to Recommend and Administer HPV Vaccines

The CDC cites in a recent report that missed clinical opportunities are the most important reason why vaccine uptake remains low in the United States. Many vaccine-eligible adolescents do not receive HPV vaccines during visits with their healthcare providers. The report states that many times, adolescents received other recommended vaccines yet did not receive the HPV vaccine. Factors contributing to providers’ hesitancy include:

Limited understanding of HPV-associated diseases and benefits of HPV vaccination, particularly for males

- Concerns about safety
- Concerns about inadequate reimbursement for vaccines
- Personal attitudes and beliefs
- Discomfort talking to parents and adolescents about a topic related to sexual behavior
- Concerns about parental resistance
- Preference for vaccinating older *vs.* younger adolescents
- Lack of time or incentives to educate parents and patients about HPV and HPV vaccines
- Lack of systems to remind providers to offer vaccines to age-eligible patients

Source: Rimer BK, Harper H, White ON. Accelerating HPV vaccine uptake: Urgency for Action to Prevent Cancer: A report to the President of the United States from the President’s Cancer Panel.

To address provider concerns, the Panel listed strategies to increase provider education on the safety and efficacy of the vaccines. Additionally, the Panel recommends the CDC to develop, test, disseminate, and evaluate the impact of integrated, comprehensive communication strategies for physicians and other health care professionals. In order to lead this effort, the Panel also suggests that more governmental funding be allocated to this cause. As the panel campaigns for the increase in demand of the HPV vaccine, they also recommend that healthcare payers be reimbursed adequately for HPV vaccination storage, administration and services.

The panel solicits the help of healthcare organizations and practices emphasizing the use of electronic health records (EHRs) and immunization information systems (IIS) to avoid missed opportunities for the HPV vaccination. This increase in funding would cover the cost of increasing vaccine uptake in adolescent males and having this information published in the Healthcare Effectiveness Data and Information Sheet (HEDIS) [16]. The HEDIS report is an important tool used in measuring the performance of health plans. Currently, HEDIS does not include information on vaccine uptake in males.

### HPV Vaccination in Males

HPV has largely been associated with a disease ridden only to women. Recent findings have suggested that HPV may also lead to HPV-related cancers and genital warts in men [17]. These include cancers of the upper aero-digestive tract and the anogenital tract, and their precursor lesions. HPV related upper aero-digestive cancers include cancer of the oral cavity, oropharynx, hypo larynx, and larynx. HPV type 16 has also been linked to the development of penile, anal, head and neck cancers [18]. Evidence from a recent study in patients with penile carcinoma (n =49) demonstrated that HPV was present in 77.5% of cases; specifically HPV types 16 and 18, which were present in 84.2% and 10.5% of cases, respectively [19]. Other cancers such as HPV-positive oropharyngeal cancer, has increased significantly in incidence over the recent decades among men. Annual numbers of overall oropharyngeal cancer cases now surpass that of cervical cancers. Unfortunately, most HPV-driven oropharyngeal cancer cases are diagnosed at an advance clinical stage (III–IV).

Increasing vaccination uptake in males is a key objective in reducing missed clinical opportunities. Given that HPV infection can result in genital warts, precancerous and cancerous lesions in both males and females, the cancer panel’s key objective in increasing vaccine uptake is increasing vaccine utilization in adolescent males, where vaccination rates remain the lowest [20]. Despite our increasing knowledge about HPV and the benefits of HPV vaccinations, its prevalence among men remains a challenge to healthcare systems around the world. Since there is no current test to detect HPV in men, the numbers of infected men is underestimated. Furthermore, most men who have a genital HPV infection may not have any signs or symptoms. To date, there is no cure for HPV; therefore, our best defense is through prevention by way of HPV education and vaccination among men. According to a national health interview survey, HPV vaccination initiation among men aged 18–26 years was 1.1%. According to this same survey, 51.8% of men aged 18–26 years had never heard of HPV, and 34.8% of men aged 18–26 years had never heard of the HPV vaccine [21]. In addition, the Healthy People initiative provides science-based, 10-year national objectives for improving the health of the U.S. population. Current Healthy People 2020 objectives include increasing HPV vaccine completion rates for females ages 13 to 15 years to 80 percent. The panel suggests that Healthy People 2020 objectives should be updated to include an HPV vaccination goal for males equivalent to that for females.

### Goal 2: Increase Parents’, Caregivers’, and Adolescents’ Acceptance of HPV Vaccines

Parents or other caregivers’ attitudes towards vaccines heavily influences whether their children receive them. One study found that parents were more likely to refuse HPV vaccines more than other recommended vaccines for a myriad of reasons. These reasons include, but are not limited to, vaccines not being needed for males, son or daughter not being sexually active, and safety concerns. Surveyed pediatricians and family practice physicians reported that parents of young adolescents get upset when vaccinations against sexually transmitted infections are suggested for their children. Some parents express their concerns that HPV vaccinations promote engaging in sexual activity. To alleviate parental concerns and ease the conversation between providers and parents about HPV vaccine uptake, the panel suggests that the CDC implement communication strategies that focus on messages and modes of delivery that are “sensitive to cultural, literacy/health literacy, and language differences of target populations”. The panel suggests that providers “resonate emotionally” with parents and caregivers, framing HPV vaccines as mechanisms of cancer prevention, while highlighting the safety and efficacy of these vaccines. They also suggest the importance of vaccinating both males and females as part of the adolescent vaccine initiative, while stressing the importance of initiation and completion of the vaccination series. In order to change the perception of HPV vaccines amongst parents and caregivers, the panel plans to utilize websites, blogs, social and print media with recruitment of vaccination proponents who are influential to target audiences to increase vaccine uptake. These efforts are believed to ease physician efforts to convey strong recommendations regarding HPV vaccination.

### Goal 3: Maximize Access to HPV Vaccination Services

Although the American Academy of Pediatrics and American Academy of Family Physicians prefer that all adolescents receive primary care, including vaccinations through primary care physicians, the panel recommends increasing the range of venues to include more providers for HPV vaccination. U.S. Schools and pharmacies are among the top two venues suggested in the proposed expansion plan. Survey results indicate that HPV vaccination in alternative settings may prove effective in increasing vaccine uptake in adolescent males, as many of the males surveyed had not had any recent health care visits.

Reports claim that schools can educate adolescents and parents on the importance of vaccination and can even provide a clinical environment to administer HPV vaccines. School-based vaccination programs have been reported to be successful in countries like Australia, the United Kingdom and Canada, which the panel suggests for adoption in the U.S. Pharmacies are highly accessible to most people in the United States, with over 275,000 pharmacists operating out of over 60,000 pharmacies nationwide. During the 2012–2013 flu season, pharmacists were influential in administering the flu vaccination as they accounted for 20 percent of vaccine received. The panel recommends that pharmacists be allowed to administer vaccines to adolescents, as many of them are prohibited from administering HPV vaccines, especially to young adolescents.

### Goal 4: Promote Global HPV Vaccine Uptake

HPV-associated cancers affect various populations worldwide, with cervical cancer being the most prevalent. The disparities in cervical cancer rates in countries such as Africa (15 times higher) *vs.* the U.S. may be attributed to the lack of cervical cancer education and screening programs. With data consistently showing the relationship between HPV types 16 and 18 and the progression of cervical cancer, the panel strongly recommends that vaccine uptake increase globally. In a global perspective, high-resourced areas such as Australia, the United Kingdom and Canada have astonishingly different health care systems than that of the United States and have done well in implementing HPV vaccination programs with rates as high as 50–85%.

Efforts to expand HPV vaccination to low- and middle-income countries (LMICs) are recognized through the efforts of the GAVI Alliance (formerly known as the Global Alliance for Vaccines and Immunization) and the Pan American Health Organization (PAHO) Revolving Fund. In 2013, GAVI launched a program that provides HPV vaccines to more than 180,000 girls in eight countries. GAVI is also helping countries like Rwanda establish a HPV vaccination program (i.e. Medical staff, supplies, distribution systems, storage management). They have also partnered with the Islamic Development Bank (IDB) to catalyze the implementation of many vaccines (including HPV vaccinations) in IDB member countries. The panel suggests that the U.S. continue to support GAVI and other world health organizations with a heavy emphasis on increasing vaccine introduction and uptake in low-income countries.

### High Priority Research to Advance Prevention of HPV-Associated Cancers

In addition to the four goals outlined in the Cancer Panel 2012–2013 report, the panel also suggested funding “high-priority” research to advance prevention of HPV-associated cancers. The panel hopes that the government would continue to fund HPV vaccine research that:

- Investigates more convenient dosing schedule for current vaccines. With hopes of producing evidence that HPV can be treated with fewer vaccine doses.
- Develops next-generation vaccines that provide better protection and/or are easier to store and administer. This would allow for changes in vaccine formulation that would increase coverage of HPV types and would allow for easier storage and management.
- Explain the natural history of or pharyngeal HPV infections. Knowledge of the pathology of or pharyngeal HPV remains to be conclusive. Research would allow for the investigation and introduction of biomarkers that aid in the recognition and diagnosis of this malignancy, which can possibly be eradicated through vaccination.
- Develop more effective ways to communicate about HPV-associated diseases and HPV vaccines through mobile health interventions. Specific strategies should be tested among different populations in accordance with sociocultural factors in order to determine the best way to convey messages regarding the HPV vaccination series.
- Determine how best to integrate HPV vaccination with cervical cancer screening. This is of particular importance because studies show that African-American females are not being screened for cervical cancer, which may account for the high incidence and mortality rates in this population.

## Discussion

The Human Papillomavirus vaccination is a novel approach to combat the incidence of HPV and its associated health outcomes in both male and female populations. The panel’s report investigates and addresses causes of low vaccine uptake not only in the U.S., but globally [22]. The low rates of uptake and completion of the HPV vaccination series in the United States may be restored by placing an emphasis on populations that HPV and cervical cancer disproportionally affect, for example, African-American females [23].

In the United States, the current cervical cancer mortality rate for Black women (BW) is almost twice that of Caucasian Women (CW) (4.3 per 100,000 (BW) *vs.* 2.2 per 100,000 (CW)). Previous researchers demonstrated that Black women experience inadequate access to preventive services or do not use these services for a variety of reasons, including but not limited to transportation issues, availability of insurance, level of income or education, fear and mistrust of the health care system [24]. Inadequate diagnostic and therapeutic care also contribute to racial differences in late-stage cervical cancer diagnoses. Some reports documented a lack of follow-up visits after a Pap test among black women, and in some cases, this occurred at a patient’s medical home. Various reports cite challenges in the patient-provider relationship that impede screening and follow-up efforts, particularly in African-American female populations. With Pap smears being paramount in the diagnosis of cervical cancer, it is imperative that future research efforts focus on ways to merge cervical cancer screenings with HPV vaccination uptake [25]. An intervention should focus on providers to improve skills required to nurture and enhance physician-patient relationship. Another study investigated factors that correlated with parental awareness of HPV vaccines using data from parents of both male and female pre-adolescents and adolescents. The study reported that their data indicated that most U.S. parents (62.6%) have heard of HPV vaccines. Multivariable results revealed that parents of children who were older, female, and insured were more likely to have heard of HPV vaccines. Parents who were female, white (non-Hispanic), English speakers, born in the U.S., married, had higher levels of education and higher incomes were also more likely to be aware of HPV vaccines. This data heavily suggests that ensuring access to quality healthcare is necessary to awareness, initiation and completion of the HPV vaccination series. As for cost and access to such vaccines, programs such as Vaccines for Children (VFC) and Merck Vaccine Patient Assistance Program (MVPAP) are available to those that have no health insurance or cannot afford the vaccines [8,26]. It is essential to address the obstacles to the uptake of the vaccines and identify and utilize solutions to promote greater awareness and access to the vaccines regardless of cultural, educational or socioeconomic backgrounds. The vaccines show potential to greatly reduce, possibly eradicating the presence of HPV-related cancers, and should be readily available to everyone globally [27,28].

## Conclusion

Accelerating HPV vaccination uptake will require the help of parents, the nation’s public health providers and other public health professionals in order to make this a high priority issue. There are 600,000 cases worldwide each year with an HPV infection that could have been prevented with the completion of the HPV vaccination series, therefore reflecting the great efforts to eradicate this disease in the future. The current vaccines; Cervarix, Gardasil, and Gardasil 9 have been scientifically proven to be effective in decreasing the amount of disparities found in underrepresented areas. With other countries achieving success in HPV vaccinations, it is proven that it can be done. Incorporating the FOUR main goals from the presidents cancer panel; reducing missed clinical opportunities to recommend and administer vaccines, increase parents, caregivers, and adolescents acceptance of HPV vaccines, maximize access to HPV vaccination services, and promoting global HPV vaccine uptake we are a step closer to eliminating Human papillomavirus which in turn will aid in eliminating cancer disparities.

## Figures and Tables

**Table 1:** Percentages of 13 to 17 Year-Old Girls Completing HPV Vaccine Series. The Center for Disease Control (CDC) estimates that efforts to increase the current HPV vaccination rates to around 80% would prevent an additional 53,000 future cervical cancer cases among 12-year-old girls or younger. Table 3, also indicate that any number under 30% is considered low uptake.

Percentage of 13-to 17-Year-Old Girls Completing HPV Vaccine Series, U.S., 2012					
Range	State	Percent	Range	State	Percent
≥50%	Rhode Island	57.7		Kentucky	34.9
	Delaware	50.4		Missouri	34.5
40–49%	Vermont	46.2		New Hampshire	34.5
	Pennsylvania	44.6		Minnesota	33.1
	Hawaii	43.4		Michigan	32.2
	Connecticut	43.6		Ohio	31.9
	Washington	43.5		South Dakota	31.8
	Massachusetts	43		New Jersey	31.6
	Maine	41.8		Alaska	31.4
	Montana	41.6		Alabama	31.1
	North Dakota	40.9		Maryland	30.9
	Louisiana	40.5		New Mexico	30.3
30–39%	New York	39.7		Texas	30.3
	Oregon	38.6		Wyoming	30.3
	District Columbia	38.5	≤29%	Georgia	29
	Oklahoma	38.4		Tennessee	28.6
	Colorado	38		Virginia	27.9
	Wisconsin	37.5		Idaho	27.8
	Nebraska	37.3		South Carolina	26.6
	Nevada	37.2		Florida	25.3
	Arizona	36.9		Kansas	25.1
	West Virginia	36.1		Utah	24.1
	North Carolina	35.5		Illinois	21.1
	California	35.8		Arkansas	18.3
	Iowa	35.6		Mississippi	12.1
	Indiana	35.2			

**Table 2:** U.S. Cancers Attributed to HPV. Adapted from The President’s Cancer Panel Report. The table demonstrates the percentage of cancers in the United States attributed to HPV. Centers for Disease Control and Prevention. Human papillomavirus-associated cancers United States, 2004–2008. (b) Gillison ML, Chaturevedi AK, Lowy DR. HPV prophylactic vaccines and the potential of noncervical cancers in both men and women. Cancer. 2008;113 (10 Suppl):3036–46 [6].

Cancer Site	Average # Cancer per Year at Site (a)	Percent Probably Caused by HPV (a)	Number Probably Caused by HPV (a)	Percent HPV Cancers Probably Caused by HPV 16 or 18 (b)	Number of Cancers per Year Probably Caused by HPV 16 or 18
Anus	4,767	93	4,500	93	4,200
Cervix	11,967	96	11,500	76	8,700
Oropharynx	11,726	63	7,400	95	7,000
Penis	1,046	36	400	87	300
Vagina	729	64	500	88	400
Vulva	3,136	51	1,600	86	1,400
Total	33,371		25,900		22,000

Credits: President Report 2012.

**Table 3:** HPV vaccines currently administered against cervical cancer causing HPV types. Gardasil, Cevarix and recently FDA approved vaccine Gardasil 9 could prevent the majority of cervical cancers if used optimally [6].

HPV TypesHPV Types Manufacturer Initial U.S Licensing	Gardasil 6, 11, 16, 18 Merck& Co. 2006	Cervarix 16, 18 GlaxoSmithKline 2009	Gardasil 9 6, 11, 16, 18, 31, 33, 45, 52, 58 Merck. 2014
Approved for prevention of	Cervical cancer and precancers, vulvar cancer and precancers, vaginal cancer and precancers, Anal cancer and precancers, genital warts	Cervical cancer and precancers	Cervical cancer and precancers, vulvar cancer and precancers, vagonal cancer and precancers, anal cancer and precancers
Approved for use in	Females (9 to 26 years old) Males (9 to 26 years old)	Females (9 to 25 years old)	Females (9 to 26 years old) Males (9 to 15 years old)

In Part Credit: President Report and update 2012–2016
